# Knowledge, attitude, practice and associated factors towards patient safety among nurses working at Asella Referral and Teaching Hospital, Ethiopia: A cross-sectional study

**DOI:** 10.1371/journal.pone.0254122

**Published:** 2021-07-01

**Authors:** Addisu Dabi Wake, Techane Sisay Tuji, Berhanu Kebe Gonfa, Eskeder Tsehayu Waldekidan, Etalem Demise Beshaw, Mohamed Ahimad Mohamed, Shawlem Tadese Geressu

**Affiliations:** 1 Nursing Department, College of Health Sciences, Arsi University, Asella, Ethiopia; 2 Inchini Primary Hospital, Oromia Regional State, Ethiopia; 3 Abomsa Primary Hospital, Oromia Regional State, Abomsa, Ethiopia; 4 Asella Referral and Teaching Hospital, Oromia Regional State, Asella, Ethiopia; 5 Gara Muleta General Hospital, Oromia Regional State, Ethiopia; 6 Gobesa Primary Hospital, Oromia Regional State, Gobesa, Ethiopia; Technion - Israel Institute of Technology, ISRAEL

## Abstract

**Background:**

Patient safety has been identified as a global priority area. It is one of the most health care challenges. There is a rising number of patients’ mortality in hospitals each year because of lapses in patient safety practice. Therefore, the purpose of the present study was to assess knowledge, attitude, practice, and associated factors towards patient safety among nurses working at Asella Referral and Teaching Hospital.

**Methods:**

Institution based cross-sectional study was conducted on 172 nurses working at Asella Referral and Teaching Hospital, Arsi Zone, Oromia Regional State, Ethiopia. The data were collected from nurses from December 28, 2020 to January 05, 2021 by using a pretested questionnaire. The data were entered into Epi-Data version 4.2.0.0 and analyzed using the SPSS version 23.0 software.

**Results:**

A total of 172 nurses were enrolled in to the study, resulting a response rate of 99.4%. The mean age of the nurses was 32.53 years. More than half 94(54.7%) of them were female. The majority 133(77.3%) of them were qualified for degrees and above. The majority 155(90.1%) of them had working experience of ≤13years. The nurse’s level of good knowledge, positive attitude, and good practice towards patient safety was 58.7% (n = 101, [95% CI; 51.7, 66.7]), 52.9% (n = 91, [95% CI; 43.6, 61.4]), and 50% (n = 86, [95% CI; 43.6, 57%]) respectively. The multivariable logistic regression analysis showed; working in the operation theatre unit [AOR = 5.01, 95% CI; 1.36, 18.46], having information on patient safety during initial education [AOR = 4.99, 95%CI; 1.87, 13.31], and having information on patient safety during continuing education [AOR = 2.85, 95% CI; 1.14, 7.12] were factors significantly associated with knowledge towards patient safety. Being male [AOR = 3.09, 95% CI; 1.38, 6.95], having working experience of >13 years [AOR = 8.37, 95% CI; 1.36, 51.70], having information on patient safety during initial education [AOR = 3.36, 95%CI; 1.11, 10.15], having information on patient safety during continuing education [AOR = 3.33, 95% CI; 1.25, 8.85], and having good Knowledge towards patient safety [AOR = 2.74, 95% CI; 1.21, 6.21] were factors significantly associated with attitude towards patient safety. Having information on patient safety during initial education [AOR = 5.35, 95%CI; 1.77, 16.17] and having a positive attitude towards patient safety [AOR = 3.02, 95% CI; 1.32, 6.91] were factors significantly associated with practice towards patient safety.

**Conclusion:**

In the present study, more than half of the nurse’s had good knowledge and positive attitude towards patient safety. However, only half of the nurses had good practice towards patient safety. Educational programs and training on patient safety may need to take place for nurses to abate these problems.

## Introduction

Patient safety can be defined as the prevention of patient injuries or adverse events that could occur during health care delivery [1]. It is one of the dimensions of care [2]. It is multifaceted, quite complex in nature and includes several key elements. The conversion of patient safety into a specific body of knowledge is relatively latest and thus it may be considered as a relatively new discipline. But, its concern is inherent to the practice of the health care professions [3]. Patient safety is a health care discipline that occurs with the developing difficulty in health care systems and the leading increase of patient harm in healthcare institutions. The purpose was to avoid and decrease the risks, errors, and harm that happen to patients during the provision of health care [4]. Besides, the major objectives of patient safety are to prevent the occurrence of preventable adverse associated with health care and to limit the impact of inevitable adverse events [3]. It is essential to offer quality critical health services. There is a clear agreement that quality health services across the globe should be effective, people centred, and safe. For the successful implementation of patient safety approaches, skilled health care professionals, leadership capacity, clear policies, data to drive safety improvements, and effective involvement of patients in their care are required [4].

Patient safety is perceived as a rising essential issue in healthcare field, and the increase in the numbers of patient safety incidents leads to a challenge for hospital management [5]. It is a worldwide problem where both economically developed and economically developing countries are affected [1]. According to the WHO (2019) report, the incidence of adverse events because of unsafe care is one of the 10 leading causes of mortality and disability globally. In economically low-income and middle-income countries, about 134 million adverse events happen in hospitals because of unsafe care, which leads to 2.6 million deaths each year. Up to 4 in 10 patients are harmed in primary and outpatient health care globally, of which about 80% of the harm is avoidable [4]. Medical errors and adverse events are a critical danger to patients globally [6]. The evidence showed that approximately 98,000 persons die per year because of the medical errors that happen in hospitals. This is more than the death from motor vehicle accidents, workplace injuries, and breast cancer. Moreover, the financial burden of human tragedy and medical error easily increases to the peak ranks of urgent and widespread community issues [7].

The advancement of medical is found to make assistance processes more and more complex, and there is usually a combination of situations that converges for errors to happen [2]. Of course, it can be at risk in both hospital and general practice settings. Even though severe patient safety incidents have been reported, quantitative researches with a large sample size of patients in general practice are rare [8]. Patient safety has obtained enlarged consideration in current years. However, generally the focus is on the magnitude of errors and adverse events rather than on practices that decrease the events. Practices with strong supportive evidence are clinical interventions that reduce the risks linked to hospitalization. Furthermore, the evidence-based strategy can support to investigate practices that are more likely to improve patient safety [9]. Patient safety and quality care are a foundation of health care systems and processes which are essentially dependent upon nurses. Nurses should play a leadership role to achieve the goals of patient safety and quality care [10]. Nurses are in the best position to improve patient safety as the largest group of healthcare providers [11]. The evidence displayed that patient safety programmes and the dissemination of study outcomes in the area have specifically supported nurses to develop safer practices [12]. Even though there are many strategies available to improve patient safety, there is no magic bullet. Thus, besides better utilization of the available methods, it is important to use new and potentially more effective methods. For instance, health professionals’ involvement in patient safety programmes is vital if improvements in patient safety are needed [13].

The study found that there was a positive correlation between the attitude of patient safety and patient safety management activities [14]. The major test in moving toward a safer healthcare system is patient safety culture, which is the prevention of danger to the patients. Safe medical practices can avoid danger to the patients. For this, healthcare professionals must have good attitudes regarding patient safety [15]. Knowledge regarding to safety in complex systems is increasing and health care is a complex system that is both growing and under pressure. Therefore, patient safety work also has to grow. Basic situations for safe performance such as management that values safety, good working circumstances, safety culture, enough staffing and competence, and equipment that facilitates safe practice are important [16].

However, the responsibility for patient safety should not be restricted to the practice of bedside nurses. Rather, patient safety should be a responsibility of all in the healthcare system [17]. The evidence showed that the middle-range theory of patient safety goal priming via safety culture communication may encourage organizations in this endeavour. Based on this theory, hospital safety culture communication stimulates a formerly held patient safety goal and raises the perceived value of actions nurses can perform to accomplish that goal. Thus, nurses consequently prioritize and are motivated to carry out tasks and risk assessments linked to accomplishing patient safety [18]. Furthermore, developing a culture of safety is a foundation component of several efforts to improve patient safety and care quality [19]. Patient safety is highly prioritized in the health care system. Since successful interprofessional collaboration is the key for patient safety, this issue should be included interprofessionally in the curriculum [20]. Additionally, developing a positive reporting culture which helps medical and healthcare workers learn from errors and decrease the risk of future adverse events, is core to fostering a culture of patient safety [21]. Ensuring safety in healthcare settings is initiating improvements both in education and clinical practice [22].

Furthermore, patient safety can be improved by standardized handovers when encouraged by technological solutions, face-to-face contact between nurses, and alongside bedside reports. However, changing nursing handover practices to augment patient safety is complex. This includes changing the culture, behaviour, and roles of a clinical nursing settings [23]. Association between patient safety culture and patient outcomes is found at a hospital and nursing unit level of analysis [24]. Furthermore, the failure to overcome the obstacles to interprofessional collaborative practice leads patients at risk for unsafe care and harmful outcomes [25]. Patient safety is vital to healthcare quality. It is significant to determine the nursing students’ safety attitudes to discover the weaknesses of a growing education programs [26]. The effectiveness of implementation is found to affect the success of patient safety and quality improvement interventions [27].

As explained above, the burden of harm during healthcare service delivery and its associated costs is increasing globally. Gaining evidence regarding the level of knowledge, attitude, practice and associated factors towards patient safety among nurses are important and significant to undertake the essential strategies required to improve the burden of harm, costs of its related issues and to enhance the quality of health care. However, there is a lack of study that has addressed knowledge, attitude, practice and associated factors towards patient safety among nurses working in the study area, even in our country, Ethiopia, there is no enough study on this critical topic. Therefore, the purpose of the present study was to determine the knowledge, attitude, practice, and associated factors towards patient safety among nurses. The result of the present study would aware the hospitals, nurses, local policy implementers, government, stakeholders, and researchers who want to conduct the study on this topic to manage and control the critical burden of incidents of harm or error during healthcare service delivery, which grows alarmingly worldwide.

## Objectives

### General objectives

- To assess the level of knowledge, attitude, practice, and associated factors towards patient safety among nurses working at Asella Referral and Teaching Hospital, Asella, Ethiopia, 2021.

### Specific objectives

- To determine the level of knowledge towards patient safety among nurses working at Asella Referral and Teaching Hospital, Asella, Ethiopia, 2021.
- To assess the level of attitude towards patient safety among nurses working at Asella Referral and Teaching Hospital, Asella, Ethiopia, 2021.
- To determine the level of practice regarding patient safety among nurses working at Asella Referral and Teaching Hospital, Asella, Ethiopia, 2021.
- To identify factors associated with knowledge towards patient safety among nurses working at Asella Referral and Teaching Hospital, Asella, Ethiopia, 2021.
- To identify factors associated with attitude towards patient safety among nurses working at Asella Referral and Teaching Hospital, Asella, Ethiopia, 2021.
- To identify factors associated with practice regarding patient safety among nurses working at Asella Referral and Teaching Hospital, Asella, Ethiopia, 2021.

## Methods

### Study area and period

The study was conducted at Asella Referral and Teaching Hospital from December 28, 2020 to January 05, 2021. Asella referral and teaching hospital is located in Asella town, Arsi zone, Oromia Regional State, Ethiopia. Asella town is located at 175 KMs far from Addis Ababa, the capital city of Ethiopia.

### Study design

Institution based Cross-sectional study was conducted on staff nurses working at Asella Referral and Teaching Hospital.

### Source population

All staff nurses working at Asella Referral and Teaching Hospital.

### Study population

Staff nurses working at Asella Referral and Teaching Hospital and who fulfilled the inclusion criteria.

### Eligibility criteria

#### Inclusion criteria

All nurses working in Asella Referral and Teaching Hospital and who were willing to participate in the study were included.

#### Exclusion criteria

The nurse who was severely ill and incompetent to participate was excluded from the study.

### Sample size determination

The prevalence of knowledge and attitude were 48.4% and 56.1%, respectively. This was taken from a study conducted in University of Gondar specialized hospital [28]. Since there was no study conducted on the practice towards patient safety in Ethiopia or other economically developing countries, we couldn’t get the prevalence of practice. The sample size was calculated by using a single proportion formula for both knowledge and attitude, by using their prevalence, a 95% confidence interval, and a margin error of 5%. Correction formula was used because the total nurses working at Asella Referral and Teaching Hospital were 264, which is less than 10,000 and a 10% nonresponse rate was added. Finally, the highest sample size taken was 173.

### Sampling techniques and procedures

Simple random sampling technique was used to select the study subjects from all staff nurses working in Asella Referral and Teaching Hospital. During this, primarily, the lists of staff nurses were obtained from the hospital. Then, based on the list obtained, a lottery method was used to select the study participants. Next, the objective of the study was explained to them. Finally, self-administered questionnaires were distributed for those nurses who were willing to participate in the study.

### Study variables

#### Dependent variables

- Knowledge towards patient safety
- Attitude towards patient safety
- Practice towards patient safety

#### Independent variables

**Sociodemographic characteristics**: age, gender, educational qualification, Ethnicity, Religion, and Marital status.

**Personal related characteristics**: working experience, working unit, work position, working hours per week, having an extra job, having information on patient safety during initial education, having information on patient safety during continuing education, and having training on patient safety.

### Operational definitions

#### Good knowledge

When nurses respond the mean or above the mean score on knowledge questions concerning to patient safety [28–31].

#### Poor knowledge

When nurses respond below the mean score on knowledge questions concerning to patient safety [28–31].

#### Positive attitude

When nurses respond the mean or above the mean score on attitude questions regarding to patient safety [28, 30, 32–35].

#### Negative attitude

When nurses respond below the mean score on the attitude questions regarding to patient safety [28, 30, 32–35].

#### Good practice

When nurses respond below the mean score on practice questions about patient safety.

#### Poor practice

When nurses respond below the mean score on practice questions about patient safety.

### Data collection instruments and procedures

The questionnaire was included socio-demographic characteristics, personal related characteristics, knowledge towards patient safety, attitude towards patient safety, and practice towards patient safety. The questionnaire was developed from the relevant literatures [5, 28, 30–35]. During this time, different experts were participated in this process. The questionnaire was prepared in English. A semistructured self-administered questionnaire was used to collect data from the selected nurses. A total of five BSc nurses’ data collectors and one MSc supervisor were recruited for the study.

### Data quality control

The quality of the data was assured by pretesting of the questionnaire and providing training on the data collection instruments and procedures. The questionnaire was pretested on 5% of the calculated sample size and given as (S1 File). Whereas, one full day duration of training was given for the data collectors concerning to the data collection instrument and procedure. The reliability of the questionnaire was checked by the reliability analysis and the value of cronbach’s alpha was suggested a reliable tool. During the data collection period, a close supervision was done by the supervisors, by supervising data collectors and checking the collected data for its completeness.

### Data processing and analysis

Data was checked, coded, and entered into Epi-Data version 4.2.0.0, and then it was exported to Statistical Package for the Social Sciences (SPSS) version 21.0 (IBM Corporation, North Castle Drive, Armonk, NY, USA) for statistical analysis.

The outcome variables were dichotomized. For instance; knowledge (poor knowledge and good knowledge), attitude (negative attitude and positive attitude), and practice (poor practice and good practice). Then these outcome variables were coded as knowledge (poor knowledge = 0 and good knowledge = 1), attitude (negative attitude = 0 and positive attitude = 1), and practice (poor practice = 0 and good practice = 1). Descriptive statistics were summarized by using tables, figures, and texts. Bivariable and multivariable logistic regression analyses were applied to identify variables associated with knowledge, attitude, and practice towards patient safety.

The crude odds ratio and adjusted odds ratio with the corresponding 95% (CI) were calculated to show the strength of the association. Whereas, the model fitness was checked by Hosmer-Lemeshow’s goodness-of-fit test for knowledge, attitude, and practice towards patient safety while the result was found to be (*p*-value = 0.375), (*p*-value = 0.575), and (*p*-value = 0.295) where (*p*-value >0.05). Finally, variables in the multivariable logistic regression with *p*-values <0.05 were considered as statistically significant.

### Ethics approval and consent to participate

Data were collected after the ethical clearance was received from Nursing department, College of Health Sciences, Arsi University. The present study was approved by Nursing department on behalf of the Institutional Ethical Review Board of Arsi University. The letter of permission was sent to Asella Referral and Teaching Hospital and permission was obtained. The nurses were informed about the objectives of the study and confidentiality issues prior to data collection. For the reason of privacy and confidentiality, personal identifiers were not used. The nurses were also informed that they have the right to withdraw from the study at any phase. After brief information was offered concerning the objectives and significance of the study for the nurses, verbal informed consent was gained. This was because of that this study didn’t include any clinical trial or no blood sample collection. Furthermore, no any other experimental activities were involved which could harm the nurses in any form. The study was done by using the self-administered questionnaire. This procedure of gaining verbal consent was approved by Nursing department on behalf of the Institutional Ethical Review Board of Arsi University.

## Result

### Sociodemographic characteristics of study participants

A total of 172 nurses were enrolled in to the study resulting a response rate of 99.4%. The mean age of the nurses was 32.53 years with the range of 24 to 57 years. The majority 89(51.7%) of the nurses were aged between 30 to 49 years. More than half 94(54.7%) of them were female. Regarding to the educational qualification, the majority 133(77.3%) of the nurses were qualified for degrees and above (Table 1).

**Table 1:** Sociodemographic characteristics of nurses’ working at Asella Referral and Teaching Hospital, Asella, Ethiopia, 2021, [n = 172].

Variables	Category	Frequency	Percent
Age in years	<35	116	67.4
	≥35	56	32.6
Gender	Male	78	45.3
	Female	94	54.7
Educational qualification	Diploma	39	22.7
	Degree and above	133	77.3
Ethnicity	Oromo	99	57.6
	Amhara	62	36
	Other	11	6.4
Religion	orthodox	51	29.7
	Muslim	74	43.0
	Protestant	41	23.8
	Other	6	3.5
Marital status	Single	35	20.3
	Married	95	55.2
	Divorced	27	15.7
	Widowed	15	8.7

### Personal related characteristics

The majority 155(90.1%) of the nurses had working experience of ≤13years. 75(43.6) Nurses had a Length of ≥50 working hours per week. Only 38(22.1%) of the nurses had training on patient safety (Table 2).

**Table 2:** Personal related characteristics of nurses’ working at Asella Referral and Teaching Hospital, Asella, Ethiopia, 2021, [n = 172].

Variables	Category	Frequency	Percent
Nurses working Experience	≤13 years	155	90.1
	>13 years	17	9.9
Nurses Working unit	Emergency	24	14.0
	Out patient	42	24.4
	Inpatient	65	37.8
	OR	41	23.8
Nurses Work position	Head nurse	13	7.6
	Staff nurse	159	92.4
Nurses Length of working hours per week	≤40	57	33.1
	41–49	40	23.3
	≥50	75	43.6
Having an extra job	Yes	31	18.0
	No	141	82.0
Having information concerning patient safety during initial education	Yes	127	73.8
	No	45	26.2
Having information concerning patient safety during continuing education	Yes	115	66.9
	No	57	33.1
Having training on patient safety	Yes	38	22.1
	No	134	77.9

### Knowledge towards patient safety

**Fig 1:**
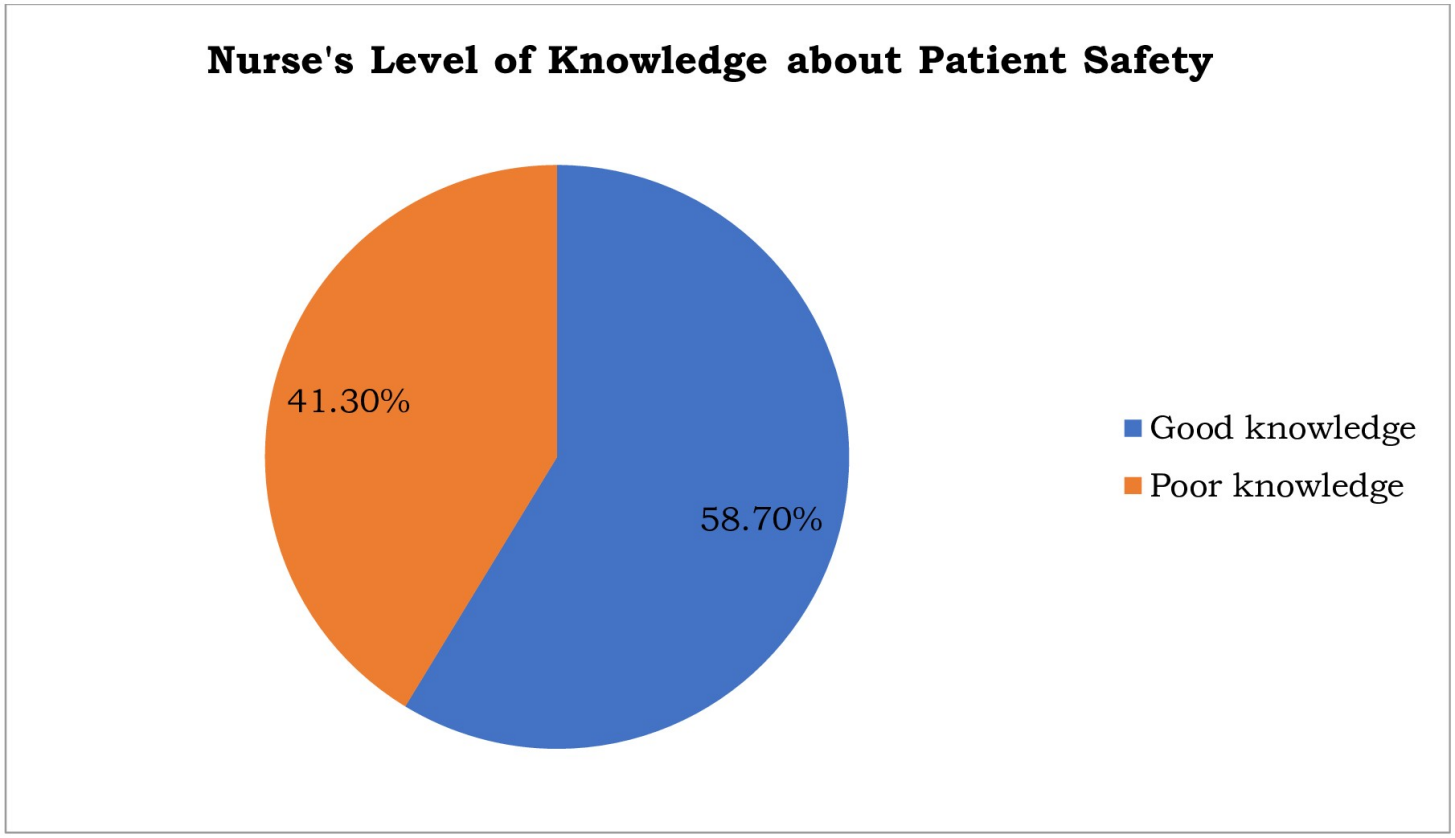
Nurse’s level of knowledge about patient safety among nurses working at Asella Referral and Teaching Hospital, Asella, Ethiopia, 2021, [n = 172].

The nurse’s level of good knowledge towards patient safety was 58.7% (n = 101, [95% CI; 51.7, 66.7]) (Fig 1).

### Attitude towards patient safety

**Fig 2:**
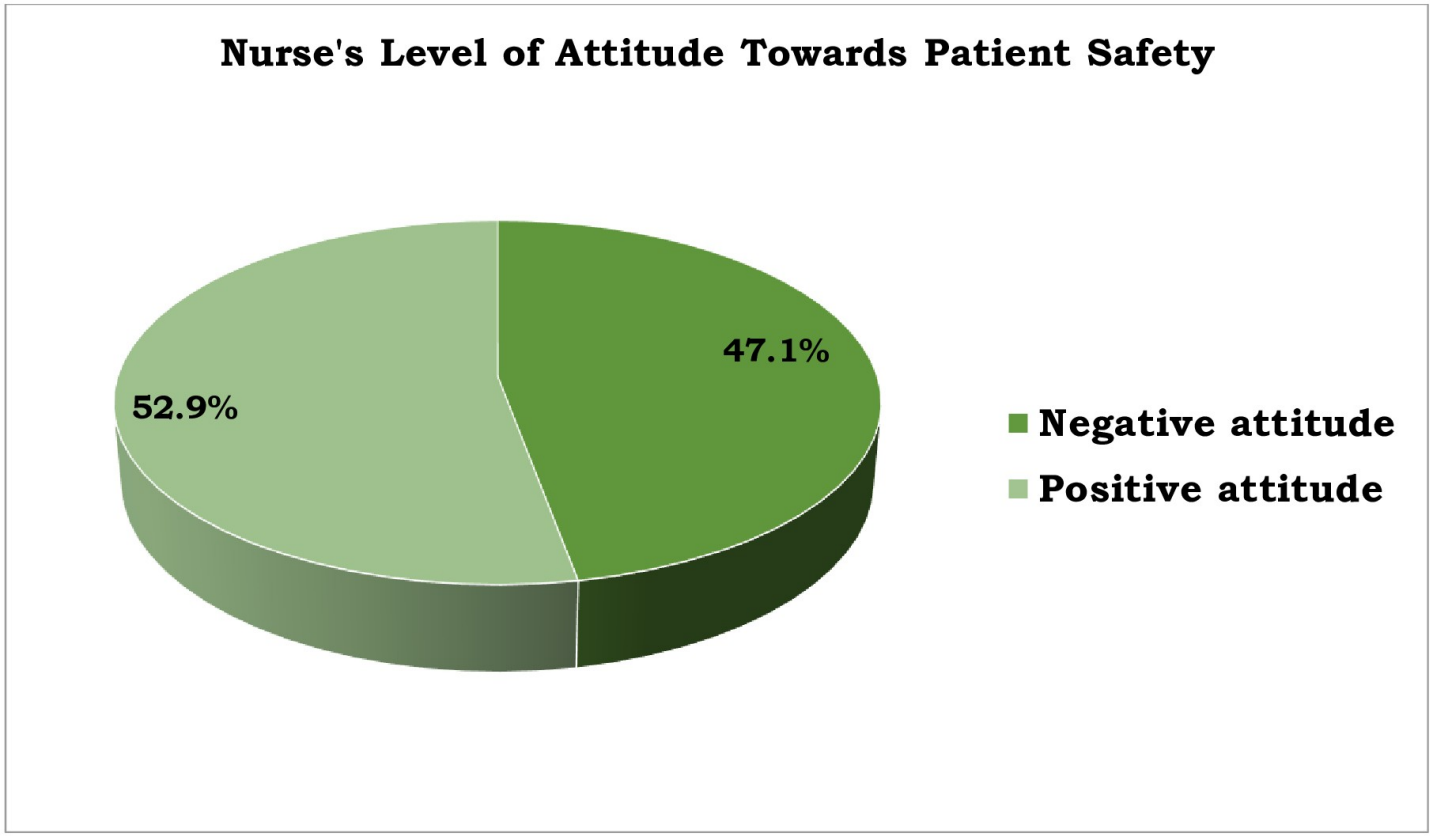
Nurse’s level of attitude about patient safety among nurses working at Asella Referral and Teaching Hospital, Asella, Ethiopia, 2021, [n = 172].

The nurse’s level of positive attitude towards patient safety was 52.9% (n = 91, [95% CI; 43.6, 61.4]) (Fig 2).

### Practice towards patient safety

**Fig 3:**
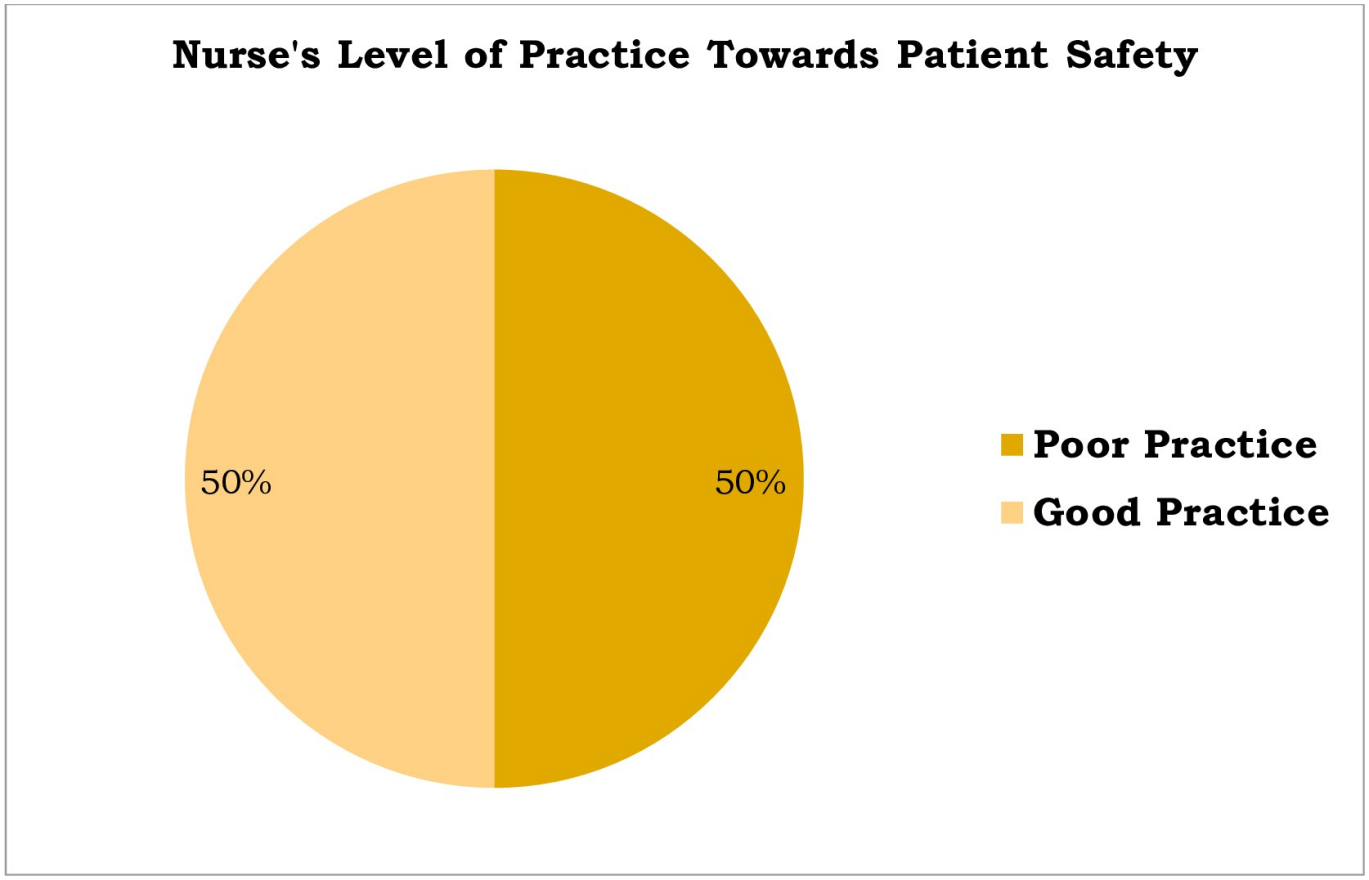
Nurse’s level of practice towards patient safety among nurses working at Asella Referral and Teaching Hospital, Asella, Ethiopia, 2021, [n = 172].

The nurse’s level of good practice towards patient safety was 50% (n = 86, [95% CI; 43.6, 57%]) (Fig 3).

### Factors associated with knowledge towards patient safety

To identify independent factors associated with knowledge towards patient safety; age, gender, educational qualification, working experience, working unit, work position, length of working hours per week, having an extra job, having information regarding patient safety during initial education, having information concerning patient safety during continuing education, and having training on patient safety were entered in to both bivariable and multivariable logistic regression analysis. However, only the working unit, having information regarding patient safety during initial education, and having information concerning patient safety during continuing education were factors significantly associated with knowledge towards patient safety.

The likelihood of having a good knowledge towards patient safety among nurses who were working in a unit of operation theatre was 5.01 times [AOR = 5.01, 95% CI; 1.36, 18.46] folds more when compared with those who were working in the emergency unit. Those participants who had information regarding patient safety during initial education were 4.99 times [AOR = 4.99, 95%CI; 1.87, 13.31] more likely to have a good knowledge towards patient safety when compared to their contraries. The odds of having a good knowledge towards patient safety among nurses who had information concerning patient safety during continuing education was 2.85 times [AOR = 2.85, 95% CI; 1.14, 7.12] higher than nurses who had not (Table 3).

**Table 3:** Bivariable and multivariable logistic regression analysis of factors associated with knowledge towards patient safety among nurses working at Asella Referral and Teaching Hospital, Asella, Ethiopia, 2021, [n = 172].

Variables	Category	Knowledge		COR (95% CI)	AOR (95% CI)	P-value
		Good	Poor			
Age in years	<35	72(62.1%)	44(37.9%)	1	1	
	≥35	29(51.8%)	27(48.2%)	0.656(0.35,1.25)	0.76(0.31,1.85)	0.541
Gender	Male	50(64.1%)	28(35.9%)	1.51(0.81,2.79)	1.44(0.67,3.09)	0.355
	Female	51(54.3%)	43(45.7%)	1	1	
Educational qualification	Diploma	23(59%)	16(41%)	1	1	
	Degree and above	78(58.6%)	55(41.4%)	0.99(0.48,2.04)	0.68(0.27,1.70)	0.410
Working experience in years	≤13	90(58.1%)	65(41.9%)	1	1	
	>13	11(64.7%)	6(35.3%)	1.32(0.47,3.76)	1.15(0.29,4.61)	0.842
Working unit	Emergency	11(45.8%)	13(54.2%)	1	1	
	Out patient	20(47.6%)	22(52.4%)	1.07(3.93,2.94)	1.31(0.37,4.61)	0.676
	Inpatient	38(58.5%)	27(41.5%)	1.66(0.65,4.27)	1.62(0.55,4.78)	0.382
	OR	32(78%)	9(22%)	4.20(1.41,12.52)	**5.01(1.36,18.46)**	**0.015**
Work position	Head nurse	7(53.8%)	6(46.2%)	1	1	
	Staff nurse	94(59.1%)	65(40.9%)	1.24(0.39,3.86)	0.53(0.12,2.61)	0.438
Length of working hours per week	≤40	35(61.4%)	22(38.6%)	1.12(0.55,2.27)	1.57(0.59,4.18)	0.369
	41–49	22(55%)	18(45%)	0.86(0.39,1.87)	1.05(0.38,2.90)	0.927
	≥50	44(58.7%)	31(41.3%)	1	1	
Having an extra job	Yes	20(64.5%)	11(35.5%)	1	1	
	No	81(57.4%)	60(42.6%)	0.74(0.33,1.67)	0.65(0.25,1.67)	0.372
Having information regarding patient safety during initial education	Yes	90(70.9%)	37(29.1%)	7.52(3.45,16.41)	**4.99(1.87,13.31)**	**0.001**
	No	11(24.4%)	34(75.6%)	1	1	
Having information concerning patient safety during continuing education	Yes	82(71.3%)	33(28.7%)	4.97(2.51,9.84)	**2.85(1.14,7.12)**	**0.025**
	No	19(33.3%)	38(66.7%)	1	1	
Having training on patient safety	Yes	25(65.8%)	13(34.2%)	1.47(0.69,3.11)	0.88(0.36,2.18)	0.782
	No	76(56.7%)	58(43.3%)	1	1	

### Factors associated with attitude towards patient safety

Likewise, to identify independent factors associated with attitude towards patient safety; age, gender, educational qualification, working experience, working unit, work position, length of working hours per week, having an extra job, having information regarding patient safety during initial education, having information concerning patient safety during continuing education, having training on patient safety and knowledge towards patient safety were variables entered in to both bivariable and multivariable logistic regression analysis. However, only gender, working experience, having information regarding patient safety during initial education, having information concerning patient safety during continuing education, and knowledge towards patient safety were factors significantly associated with attitude towards patient safety.

The odds of having a positive attitude towards patient safety among nurses who were male was 3.09 times [AOR = 3.09, 95% CI; 1.38, 6.95] higher than nurses who female. The likelihood of having a positive attitude towards patient safety among nurses who had a working experience of >13 years were 8.37 times [AOR = 8.37, 95% CI; 1.36, 51.70] folds more when compared with nurses who had a working experience of ≤13 years.

Those nurses who had information regarding patient safety during initial education were 3.36 times [AOR = 3.36, 95%CI; 1.11, 10.15] more likely to have a positive attitude towards patient safety when compared to their contraries. The odds of having a positive attitude towards patient safety among nurses who had information concerning patient safety during continuing education was 3.33 times [AOR = 3.33, 95% CI; 1.25, 8.85] higher than nurses who had not. The likelihood of having a positive attitude towards patient safety among nurses who had a good knowledge towards patient safety were 2.74 times [AOR = 2.74, 95% CI; 1.21, 6.21] folds more when compared with nurses who had poor knowledge towards patient safety (Table 4).

**Table 4:** Bivariable and multivariable logistic regression analysis of factors associated with attitude towards patient safety among nurses working at Asella Referral and Teaching Hospital, Asella, Ethiopia, 2021, [n = 172].

Variables	Category	Attitude		COR (95% CI)	AOR (95% CI)	P-value
		Positive	Negative			
Age in years	<35	63(54.3%)	53(45.7%)	1.19(0.63,2.25)	1.45(0.58,3.61)	0.424
	≥35	28(50.0%)	28(50.0%)	1	1	
Gender	Male	50(64.1%)	28(35.9%)	0.43(0.23,0.80)	**3.09(1.38,6.95)**	**0.006**
	Female	41(43.6%)	53(56.4%)	1	1	
Educational qualification	Diploma	22(56.4%)	17(43.6%)	1	1	
	Degree and above	69(51.9%)	64(48.1%)	0.83(0.41,1.71)	0.53(0.21,1.37)	0.189
Working experience in years	≤13	77(49.7%)	78(50.3%)	1	1	
	>13	14(82.4%)	3(17.6%)	4.73(1.31,17.11)	**8.37(1.36,51.70)**	**0.022**
Working unit	Emergency	10(41.7%)	14(58.3%)	1	1	
	Out patient	22(52.4%)	20(47.6%)	1.54(0.56,4.24)	3.30(0.81,13.46)	0.096
	Inpatient	35(53.8%)	30(46.2%)	1.63(0.63,4.21)	1.44(0.45,4.59)	0.536
	OR	24(58.5%)	17(41.5%)	1.98(0.71,5.49)	1.49(0.41,5.42)	0.545
Work position	Head nurse	6(46.2%)	7(53.8%)	1	1	
	Staff nurse	85(53.5%)	74(46.5%)	1.34(0.43,4.17)	0.96(0.19,4.65)	0.960
Length of working hours per week	≤40	30(52.6%)	27(47.4%)	1	1	
	41–49	20(50%)	20(50%)	0.9(0.40,2.02)	1.14(0.39,3.28)	0.806
	≥50	41(54.7%)	34(45.3%)	1.09(0.54,2.17)	1.29(0.48,3.50)	0.615
Having an extra job	Yes	18(58.1%)	13(41.9%)	1.29(0.59,2.83)	1.32(0.49,3.56)	0.584
	No	73(51.8%)	68(48.2%)	1	1	
Having information regarding patient safety during initial education	Yes	82(64.6%)	45(35.4%)	7.29(3.22,16.48)	**3.36(1.11,10.15)**	**0.032**
	No	9(20%)	36(80%)	1	1	
Having information concerning patient safety during continuing education	Yes	77(67%)	38(33%)	6.22(3.04,12.75)	**3.33(1.25,8.85)**	**0.016**
	No	14(24.6%)	43(75.4%)	1	1	
Having training on patient safety	Yes	24(63.2%)	14(36.8%)	0.58(0.28,1.22)	1.01(0.40,2.53)	0.988
	No	67(50%)	67(50%)	1	1	
Knowledge towards patient safety	Good	69(68.3%)	32(31.7%)	4.8(2.49,9.24)	**2.74(1.21,6.21)**	**0.016**
	Poor	22(31%)	49(69%)	1	1	

### Factors associated with practice towards patient safety

Similar to that of knowledge and attitude; to identify independent factors associated with practice towards patient safety; age, gender, educational qualification, working experience, working unit, work position, length of working hours per week, having an extra job, having information regarding patient safety during initial education, having information concerning patient safety during continuing education, having training on patient safety, knowledge towards patient safety, and attitude towards patient safety were variables entered in to both bivariable and multivariable logistic regression analysis. However, only having information regarding patient safety during initial education and attitude towards patient safety were factors significantly associated with practice towards patient safety.

Those nurses who had information regarding patient safety during initial education were 5.35 times [AOR = 5.35, 95%CI; 1.77, 16.17] more likely to have a good practice towards patient safety when compared to their contraries. The likelihood of having a good practice towards patient safety among nurses who had a positive attitude towards patient safety were 3.02 times [AOR = 3.02, 95% CI; 1.32, 6.91] folds more when compared with nurses who had a negative attitude towards patient safety (Table 5).

**Table 5:** Bivariable and multivariable logistic regression analysis of factors associated with practice towards patient safety among nurses working at Asella Referral and Teaching Hospital, Asella, Ethiopia, 2021, [n = 172].

Variables	Category	Practice		COR (95% CI)	AOR (95% CI)	P-value
		Good	Poor			
Age in years	<35	59(50.9%)	57(49.1%)	1.11(0.59,2.11)	1.03(0.41,2.58)	0.943
	≥35	27(48.2%)	29(51.8%)	1	1	
Gender	Male	37(47.4%)	41(52.6%)	1	1	
	Female	49(52.1%)	45(47.9%)	1.21(0.66,2.20)	1.48(0.67,3.29)	0.337
Educational qualification	Diploma	21(53.8%)	18(46.2%)	1	1	
	Degree and above	65(48.9%)	68(51.1%)	0.82(0.40,1.68)	0.81(0.31,2.12)	0.670
Experience in years	≤13	75(48.4%)	80(51.6%)	1	1	
	>13	11(64.7%)	6(35.3%)	1.96(0.69,5.55)	1.16(0.26,5.11)	0.846
Working unit	Emergency	9(37.5%)	15(62.5%)	1	1	
	Out patient	17(40.5%)	25(59.5%)	1.13(0.40,3.18)	1.25(0.34,4.63)	0.734
	Inpatient	40(61.5%)	25(38.5%)	2.67(1.02,7.0)	2.80(0.88,8.93)	0.082
	OR	20(48.8%)	21(51.2%)	1.59(0.57,4.44)	1.6(0.47,5.49)	0.455
Work position	Head nurse	5(38.5%)	8(61.5%)	1	1	
	Staff nurse	81(50.9%)	78(49.1%)	1.66(0.52,5.29)	1.07(0.22,5.29)	0.932
Working hours per week	≤40	29(50.9%)	28(49.1%)	1	1	
	41–49	14(35.0%)	26(65.0%)	0.52(0.23,1.19)	0.48(0.17,1.31)	0.151
	≥50	43(57.3%)	32(42.7%)	1.29(0.65,2.59)	0.79(0.31,2.0)	0.612
Having an extra job	Yes	13(41.9%)	18(58.1%)	1	1	
	No	73(51.8%)	68(48.2%)	1.49(0.68,3.26)	2.21(0.84,5.84)	0.111
Having information regarding patient safety during initial education	Yes	79(62.2%)	48(37.8%)	8.94(3.69,21.59)	**5.35(1.77,16.17)**	**0.003**
	No	7(15.6%)	38(84.4%)	1	1	
Having information concerning patient safety during continuing education	Yes	71(61.7%)	44(38.3%)	4.52(2.25,9.09)	1.23(0.47,3.19)	0.671
	No	15(26.3%)	42(73.7%)	1	1	
Having training on patient safety	Yes	25(65.8%)	13(34.2%)	2.3(1.09,4.88)	1.97(0.78,4.98)	0.153
	No	61(45.5%)	73(54.5%)	1	1	
Knowledge towards patient safety	Good	59(58.4%)	42(41.6%)	2.29(1.23,4.26)	0.93(0.41,2.14)	0.867
	Poor	27(38%)	44(62%)	1	1	
Attitude towards patient safety	Positive	60(65.9%)	31(34.1%)	4.09(2.17,7.74)	**3.02(1.32,6.91)**	**0.009**
	Negative	26(32.1%)	55(67.9%)	1	1	

## Discussion

The present study was done to assess the knowledge, attitude, practice, and associated factors towards patient safety among nurses. This is because; knowing the levels of knowledge, attitude, practice, and associated factors towards patient safety is a cornerstone for the management and control of the morbidity and mortality associated with errors and harms during medical care services.

### Knowledge towards patient safety

The present study showed that the nurses’ level of good knowledge towards patient safety was 58.7% (n = 101, [95% CI; 51.7, 66.7]). The present study finding was lower when compared with a study conducted in Public University of Paraná, Brazil, which reported the knowledge of nurses’ towards patient safety as 89.8% [29]. The variation might be due to that the differences in socio-economic characteristics. The present study finding was consistent with the study conducted in Saudi Arabia which reported the self- rated good level of knowledge on patient safety 52.7% [30].

The present study finding was higher when compared with a study conducted in Urmia University of Medical Sciences, West Azerbaijan province, Iran, where the level of good knowledge towards patient safety was 50% [31]. The variation might be due to that the difference in the study population. A study conducted in Urmia University of Medical Sciences, West Azerbaijan province, Iran, was done among the students studying medicine, nursing, and midwifery. However, the present study was conducted among the staff nurses working in hospital. The present study finding was also higher when compared with a study conducted in University of Gondar specialized hospital where nurses’ level of good knowledge towards patient safety was 48.4% [28]. This might be due to the differences in sample size, and the number of nurses who had taken the training on patient safety (15.5% for the study conducted in University of Gondar specialized hospital Vs 22.1% for the present study).

### Attitude towards patient safety

The present study showed that the nurse’s level of positive attitude towards patient safety was 52.9% (n = 91, [95% CI; 43.6, 61.4]). The present study finding was lower than the study conducted in Manisa, Turkey, where the health professionals’ attitude towards patient rights and patient safety was (100%) [33]. The variation might be due to that the difference in sample size (318) and the questionnaires incorporated both patient rights and patient safety for the study conducted in Manisa, Turkey. The present study finding was also lower than the study conducted in University of Gondar where the level of positive attitude of patient safety was (84.33%) [32]. The possible justification might be due to that the differences in sample size and the study population while 83 and pharmacy students were the sample size and the study population, respectively, for the study done in University of Gondar.

The present study finding was consistent when compared with another study conducted in University of Gondar specialized hospital where nurses’ level of positive attitude towards patient safety was 56.1% [28]. The present study finding was also consistent with the study conducted in Saudi Arabia which reported the self-rated positive level of attitude toward patient safety (60.7%) [30].

The present study finding was also consistent with the study conducted in Central Saudi Arabia which reported the overall perception of patient safety among participants as 57.9%) [34]. Whereas, the present study finding was higher when compared to the conducted in Jimma Zone Public Hospitals where the overall perception of patient safety was found to be 36.77% [35]. The variation might be due to that the duration since the study conducted in which the study of Jimma Zone Public Hospitals was conducted from March 15 to April 9, 2017.

### Practice towards patient safety

The nurse’s level of good practice towards patient safety was 50% (n = 86, [95% CI; 43.6, 57%]).

### Factors associated with knowledge towards patient safety

The likelihood of having a good knowledge towards patient safety among nurses who were working in the unit of operation theatre were 5.01 times [AOR = 5.01, 95% CI; 1.36, 18.46] folds more when compared with those who were working in an emergency unit. The possible justification could be that relatively the operation theatre is the unit where the critical activities would be held and also requires more concern for patient safety like by using a patient safety check list. Besides, since this unit is more considerable, there might be more update for nurses regarding patient safety and overall, this would improve the knowledge of nurses towards patient safety.

Those participants who had information regarding patient safety during initial education were 4.99 times [AOR = 4.99, 95%CI; 1.87, 13.31] more likely to have a good knowledge towards patient safety when compared to their contraries. This finding was supported by a study conducted in multidisciplinary hospitals in Western Lithuania [36]. This could be because of that it is true that having information would improve the knowledge level of the nurses. This is why information dissemination is a significant approach of improving the knowledge level of healthcare providers.

The odds of having a good knowledge towards patient safety among nurses who had information concerning patient safety during continuing education was 2.85 times [AOR = 2.85, 95% CI; 1.14, 7.12] higher than nurses who had not. This finding was supported by a study conducted in University of Gondar specialized hospital and multidisciplinary hospitals in Western Lithuania [28, 36]. The possible justification could be that information has the power to minimize the confusion. This means that having information about patient safety would have the substantial effect on the knowledge regarding patient safety.

### Factors associated with attitude towards patient safety

The odds of having a positive attitude towards patient safety among nurses who were male was 3.09 times [AOR = 3.09, 95% CI; 1.38, 6.95] higher than nurses who female. The likelihood of having a positive attitude towards patient safety among nurses who had a working experience of >13 years were 8.37 times [AOR = 8.37, 95% CI; 1.36, 51.70] folds more when compared with nurses who had a working experience of ≤13 years. Those nurses who had information regarding patient safety during initial education were 3.36 times [AOR = 3.36, 95%CI; 1.11, 10.15] more likely to have a positive attitude towards patient safety when compared to their contraries. This finding was supported by a study conducted in University of Gondar specialized hospital [28].

The odds of having a positive attitude towards patient safety among nurses who had information concerning patient safety during continuing education was 3.33 times [AOR = 3.33, 95% CI; 1.25, 8.85] higher than nurses who had not. This finding was supported by a study conducted in University of Gondar specialized hospital [28]. The likelihood of having a positive attitude towards patient safety among nurses who had a good knowledge towards patient safety were 2.74 times [AOR = 2.74, 95% CI; 1.21, 6.21] folds more when compared with nurses who had poor knowledge towards patient safety. This finding was supported by a study conducted in University of Gondar specialized hospital [28].

### Factors associated with practice towards patient safety

Those nurses who had information regarding patient safety during initial education were 5.35 times [AOR = 5.35, 95%CI; 1.77, 16.17] more likely to have a good practice towards patient safety when compared to their contraries. The possible justification could be that since having information could improve the knowledge level, this by in turn would affect the practice. Having information regarding patient safety would affect the practice towards patient safety because of the nurses have full of information they are expected to practice.

The likelihood of having a good practice towards patient safety among nurses who had a positive attitude towards patient safety were 3.02 times [AOR = 3.02, 95% CI; 1.32, 6.91] folds more when compared with nurses who had a negative attitude towards patient safety. This might be due to that having a positive attitude towards patient safety would affect the practice of patient safety in the positive direction.

## Conclusion

In the present study, more than half of the nurses had good knowledge and positive attitude towards patient safety. Only half of the nurses had good practice towards patient safety. The multivariable logistic regression analysis showed the working unit, having information on patient safety during initial education, and having information on patient safety during continuing education were factors significantly associated with knowledge towards patient safety. Gender, working experience, having information on patient safety during initial education, having information on patient safety during continuing education, and knowledge towards patient safety were factors significantly associated with attitude towards patient safety. Having information on patient safety during initial education and attitude towards patient safety were factors significantly associated with practice towards patient safety.

Moreover, the present study offers significant evidence to support of public health and to avoid errors and harm-related morbidity and mortality during medical service. Besides, it would give the fundamental information clinically for the hospital to focus on the patient safety and also would support them to propose methods to prevent the problem. Lastly, we suggest educational programs and training on patient safety may need to take place for nurses to abate these problems.

## Limitations of the present study

The accomplishment of the present study was not without limitations. Despite this is a critical topic, the level of knowledge, attitude, practice, and associated factors towards patient safety among nurses were not adequately assessed in Ethiopia, even in different countries globally. This has affected the discussion section of the present study. However, hopefully this study could minimize such problem being a baseline for other researchers who will be willing to undertake the study on this topic.

## Supporting information

S1 File Questionaries. (DOCX)
