# Supplementary material for: Knowledge, attitude, practice and associated factors towards patient safety among nurses working at Asella Referral and Teaching Hospital, Ethiopia: A cross-sectional study
Source: PLoS One. 2021 Jul 1;16(7):e0254122. doi: 10.1371/journal.pone.0254122 (PMC8248719; doi:10.1371/journal.pone.0254122)
Supplement: S1 File — (DOCX) [file pone.0254122.s001.docx]

S1 File. Questionaries

Regarding to the survey or questionnaire used in the study, anyone can replicate it. Basically, the questionnaire was developed in English language as part of this study and it is not under a copyright more restrictive than CC-BY. The copy is included below in English language as a supporting information.

| Part I: Socio-demographic characteristics of respondents | | |
| --- | --- | --- |
| **S. N** | **Questions** | **Response** |
| 101 | Age | ___________ (years) |
| 102 | Gender | 1. Male 2. Female |
| 103 | Educational qualification | 1. Diploma 2. Degree 3. Masters and above |
| 104 | Ethnicity | 1. Oromo 2. Amhara 3. Other |
| 105 | Religion | 1. Orthodox 2. Muslim 3. Protestant 4. Other |
| 106 | Marital status | 1. Single 2. Married 3. Divorced 4. Widowed |

| **Part II: Personal Related Characteristics** | | |
| --- | --- | --- |
| **S.N** | **Questions** | **Response** |
| 201 | Years of experience | ____________(years) |
| 202 | Working unit | 1. Inpatient 2. Outpatient 3. Emergency 4. ICU 5. OR |
| 203 | Work position | 1. Head nurse 2. Staff nurse |
| 204 | Working hours per weeks | ________(hours) |
| 205 | Having extra job | 1. Yes 2. No |
| 206 | Having an information about patient safety during initial education. | 1. Yes 2. No |
| 207 | Having an information about patient safety during continuing education. | 1. Yes 2. No |
| 208 | Having a training about patient safety. | 1. Yes 2. No |

| Part III: Knowledge related questionnaires | | |
| --- | --- | --- |
| **S. N** | **Questions** | **Response** |
| 301 | The clinical environment can be a cause of errors. | 1. Yes 2. No |
| 302 | The medical errors are a sign of incompetence. | 1. Yes 2. No |
| 303 | The key to patient safety strategies is set by the national health surveillance. | 1. Yes 2. No |
| 304 | Patients have an important role in preventing errors. | 1. Yes 2. No |
| 305 | Human error is inevitable. | 1. Yes 2. No |
| 306 | An adverse event is an event that affected the patient. | 1. Yes 2. No |
| 307 | Patient safety is the characteristic of highly reliable health care Organization. | 1. Yes 2. No |
| 308 | The key dimension of patient safety is a culture. | 1. Yes 2. No |
| 309 | A mistake is a failure to execute an action plan as intended or an implementation of the wrong plan. | 1. Yes 2. No |
| 310 | There is contributing factors to the occurrence of clinical errors. | 1. Yes 2. No |
| 311 | There should be a next step to be done after the occurrence of an error. | 1. Yes 2. No |

| Part IV: attitude related questionnaires | | | | | | |
| --- | --- | --- | --- | --- | --- | --- |
| **S. N** | **Questions** | **Response** | | | | |
|  |  | Strongly Disagree | Disagree | Neutral | Agree | Strongly Agree |
| 401 | Nurse input is well received in this clinical area |  |  |  |  |  |
| 402 | In this clinical area, it is easy to speak up if I perceive a problem with patient care |  |  |  |  |  |
| 403 | Disagreements in this clinical area are resolved appropriately |  |  |  |  |  |
| 404 | I have the support I need from other personnel to care for patients |  |  |  |  |  |
| 405 | It is easy for personnel here to ask questions when there is something that they do not understand |  |  |  |  |  |
| 406 | The health care workers here work together as a well-coordinated team |  |  |  |  |  |
| 407 | I would feel safe being treated here as a patient |  |  |  |  |  |
| 408 | Medical errors are handled appropriately in this clinical Area |  |  |  |  |  |
| 409 | I receive appropriate feedback about my performance |  |  |  |  |  |
| 410 | I know the proper channels to direct questions regarding patient safety |  |  |  |  |  |
| 411 | In this clinical area it is easy to discuss errors |  |  |  |  |  |
| 412 | I am encouraged by my colleagues to report any patient safety concerns |  |  |  |  |  |
| 413 | This clinical area makes it easy to learn from the errors of others |  |  |  |  |  |
| 414 | Management does not knowingly compromise patient safety |  |  |  |  |  |
| 415 | Fatigue impairs my performance during emergency situations. |  |  |  |  |  |

| **Part V: Practice related questionnaires** | | |
| --- | --- | --- |
| **S. N** | **Questions** | **Response** |
| 501 | Is there Teamwork within the unit? | 1. Yes 2. No |
| 502 | Is that Teamwork across the unit? | 1. Yes 2. No |
| 503 | Is that supervisor’s expectation & action promoting patient safety? | 1. Yes 2. No |
| 504 | Is there Feedback and communications about Error? | 1. Yes 2. No |
| 505 | Is there non-punitive response to error? | 1. Yes 2. No |
| 506 | Does hospital management support for patient safety? | 1. Yes 2. No |
| 507 | Does hospital’s handoffs and transfer patient? | 1. Yes 2. No |
| 508 | Is there an Organizational learning/continuous improvement? | 1. Yes 2. No |
| 509 | Is that composite percentage of perception of patient safety practice? | 1. Yes 2. No |
